# Electrically Tunable Broadband Terahertz Absorption with Hybrid-Patterned Graphene Metasurfaces

**DOI:** 10.3390/nano8080562

**Published:** 2018-07-24

**Authors:** Longfang Ye, Xin Chen, Guoxiong Cai, Jinfeng Zhu, Na Liu, Qing Huo Liu

**Affiliations:** 1 Institute of Electromagnetics and Acoustics, and Department of Electronic Science, Xiamen University, Xiamen 361005, China; xchen34320161153059@stu.xmu.edu.cn (X.C.); gxcai8303@xmu.edu.cn (G.C.); nanoantenna@hotmail.com (J.Z.); liuna@xmu.edu.cn (N.L.); 2 Shenzhen Research Institute of Xiamen University, Shenzhen 518057, China; 3 Department of Electrical and Computer Engineering, Duke University, Durham, NC 27708, USA; qhliu@duke.edu

**Keywords:** terahertz, graphene, broadband absorber, surface plasmons, metasurface

## Abstract

We numerically demonstrate a broadband terahertz (THz) absorber that is based on a hybrid-patterned graphene metasurface with excellent properties of polarization insensitivity, wide-angle, and active tunability. Our design is made up of a single-layer graphene with periodically arranged hybrid square/disk/loop patterns on a multilayer structure. We find that broadband absorption with 90% terahertz absorbance and the fractional bandwidth of 84.5% from 1.38 THz to 3.4 THz can be achieved. Because of the axisymmetric configuration, the absorber demonstrates absolute polarization independence for both transverse electric (TE) and transverse magnetic (TM) polarized terahertz waves under normal incidence. We also show that a bandwidth of 60% absorbance still remains 2.7 THz, ranging from 1.3 THz to 4 THz, for a wide incident angle ranging from 0° to 60°. Finally, we find that by changing the graphene Fermi energy from 0.7 eV to 0 eV, the absorbance of the absorbers can be easily tuned from more than 90% to lower than 20%. The proposed absorber may have promising applications in terahertz sensing, detecting, imaging, and cloaking.

## 1. Introduction

With the rapid development of terahertz technology, terahertz absorbers have attracted increasing attention in recent years [1]. Terahertz absorbers are one of the most important terahertz devices, having extensive applications in terahertz sensing, imaging, and detecting [2,3,4,5]. After Landy et al. creatively proposed a perfect metamaterial absorber in 2008 [6], various kinds of absorbers have been proposed and studied. For example, absorbers with periodic arrays of metallic square ring/cross/hash-shaped resonators, circular split rings, combined metamaterial structures, 3D TiN nanopillars, and all-dielectric metasurfaces were proposed to achieve single narrowband, dual/multi-narrowband, and broadband perfect absorption [3,7,8,9,10,11,12,13,14,15]. However, these metamaterial or metasurface absorbers consist of normal metals and dielectric materials, which have the inherent drawback of non-adjustability after fabrication. It would be very worthwhile to make these absorbers with both broadband and tunable absorption properties, which are especially valuable for optoelectronic devices and electromagnetic spectrum detection, modulation, and harvest applications.

Surface plasmons are the electromagnetic waves coupled to collective excitations that usually exist at the metal/dielectric interface, which have extensive promising applications in optics, microscopy, biosensing, etc. [16]. Graphene is a single-layered two-dimensional material with unique features of mechanical, chemical, and electrically tunable properties, making it a viable candidate for various tunable devices. Graphene supports surface plasmons in the terahertz and infrared ranges [16,17], demonstrating wide applications in the tunable terahertz and infrared plasmonic devices, such as waveguides [18], modulators [19], and absorbers [20]. The performance of graphene-based devices can be manipulated by changing the Fermi energy via chemical doping or electrostatic doping without changing the structure of the devices. However, notably, a single-layer graphene is almost transparent owing to its relatively low carrier concentration [21,22]. To enhance the absorption of incident waves, a variety of resonance-relying narrowband absorbers with periodic structures such as patterned graphene disks [20,23], ribbons [24], and cross-shaped fishnets [25], have been proposed. Moreover, to increase the absorption bandwidth, the graphene-based tunable absorbers with the isolated multiresonator [26], non-structured graphene-dielectric brick structures [27], multilayer graphene structures [28,29], and structured graphene patterns have been investigated [30,31,32]. However, the fractional bandwidth of the 90% absorbance for these single-layered nonstructured graphene absorbers remains relatively low (<50%). Although the fractional bandwidth of the absorbers with isolated multiresonanator or multilayered graphene structures can be further enhanced, most of them suffer from some drawbacks, including difficulty in absorption tuning via biasing, polarization sensitivity and angle dependence of the incident waves. Therefore, it is advantageous to further investigate new graphene-based terahertz absorbers with a broader bandwidth, as well as better polarization insensitivity, omnidirectionality, and flexible tunability.

In this study, we propose a novel absorber that is made up of a single-layer periodically hybrid-patterned graphene on a multilayer structure for wide-angle and polarization-insensitive broadband terahertz absorption with flexibly active tunability. The patterned graphene is an electric continuously net-shaped structure with periodically arranged hybrid squares and disks that are connected by graphene loops, which enables the broadband terahertz absorption and the possibility of absorption tuning using a simple gating layer structure. We first demonstrate the structure design of the proposed tunable polarization-insensitive terahertz absorber. Then, we investigate the broadband terahertz absorption properties of the absorber under normal and oblique incidence for both TE and TM polarizations, as well as compare the absorption spectra and electric field distributions among the absorbers with different graphene patterns. To better understand the mechanism of the broadband absorption properties, we further analyze the field distributions of two graphene in-plane resonant mode peaks at 1.6 THz and 3.2 THz and the influence of the geometric parameters on the absorption spectra. The analysis reveals that 90% terahertz absolute (fractional) absorbance bandwidths of 1.41 THz (64%) and 0.72 THz (30%) can be obtained for the absorbers with only the square-patterned and only the disk-patterned graphene layer, respectively. However, the bandwidth can be significantly increased to 2.02 THz (84.5%) for the proposed absorber with a hybrid square/disk/loop patterned graphene. Because of the axisymmetric configuration, the absorber also has excellent properties of absolute polarization insensitivity for both TE and TM polarized terahertz waves under normal incidence. Its bandwidth of 60% terahertz absorbance still remains 2.7 THz, ranging from 1.3 THz to 4 THz for a wide incident angle ranging from 0° to 60° for both polarizations. Finally, the active tunability of broadband terahertz absorption is studied. By controlling the graphene Fermi energy from 0.7 eV to 0 eV, the absorbance of the absorbers can be easily tuned from higher than 90% to lower than 20%. The proposed absorber may have promising applications in terahertz sensing, detecting, imaging, and cloaking.

## 2. The Absorber Design

**Figure 1:**
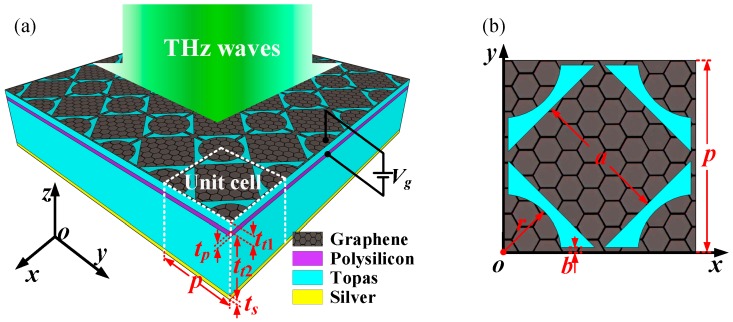
(**a**) Schematic of the proposed tunable polarization-insensitive broadband terahertz absorber, which is a multilayer structure with a single-layer patterned graphene-dielectric spacer-gating layer-dielectric spacer-metallic reflecting plate from the top to the bottom; (**b**) Top view of the absorber unit cell. The graphene layer has a periodically arranged hybrid square/disk/loop structure for electric-continuity and gating convenience, and the dimensions of the absorber are set as *p* = 40 μm, *a* = $20\sqrt{2}$ μm, *r* = 11 μm, *b* = 250 nm, *t_g_* = 0 nm, *t_t_*_1_ = 20 nm, *t_p_* = 20 nm, *t_t_*_2_ = 20.96 μm, *t_s_* = 500 nm.

The proposed electrically tunable broadband terahertz absorber using a hybrid-patterned graphene metasurface and its unit cell are schematically depicted in Figure 1. The absorber is a multilayer structure with a single-layer patterned graphene-dielectric spacer-gating layer-dielectric spacer-metallic reflecting plate from the top to the bottom. The top layer is a net-shaped patterned graphene sheet with periodically arranged hybrid squares and disks that are connected with graphene loops for electric-continuity and gating convenience. In the terahertz region, the surface conductivity of the graphene is calculated using the Kubo formula as *σ_g_* = *σ_intra_* + *σ_inter_* (Unit: S) with the intraband and interband contributions [33,34,35],

(1) $$\sigma_{intra}(\omega ,\mu_{c},\Gamma ,T)=\frac{je^{2}}{\pi \hbar^{2}\left(\omega -j2\Gamma \right)}\overset{\infty }{\underset{0}{\int }}\left(\frac{\partial f_{d}\left(\xi ,\mu_{c},T\right)}{\partial \xi }-\frac{\partial f_{d}\left(-\xi ,\mu_{c},T\right)}{\partial \xi }\right)\xi d\xi ,$$

(2) $$\sigma_{inter}(\omega ,\mu_{c},\Gamma ,T)=-\frac{je^{2}\left(\omega -j2\Gamma \right)}{\pi \hbar^{2}}\overset{\infty }{\underset{0}{\int }}\frac{f_{d}\left(-\xi ,\mu_{c},T\right)-f_{d}\left(\xi ,\mu_{c},T\right)}{{\left(\omega -j2\Gamma \right)}^{2}-4\xi /\hbar^{2}}d\xi ,$$

where $f_{d}(\xi ,\mu_{c},T)={\left(e^{\left(\xi -\mu_{c}\right)/k_{B}T}+1\right)}^{-1}$ is the Fermi-Dirac distribution, *ω* is the radian frequency, *μ_c_* is the chemical potential or Fermi level, *T* is the temperature, *Γ* is the phenomenological scattering rate, and *Γ* = 2*τ*^−1^, *τ* is the relaxation time, *e* is the charge of an electron, *ξ* is energy, *ћ* is the reduced Plank’s constant, and *k_B_* is the Boltzmann’s constant. The dielectric spacer layers are assumed to be a lossless polyethylene cyclic olefin copolymer (Topas) with a permittivity of *ε_d_* = 2.35 [32,36,37] below the graphene sheet and upon the metallic layer. By taking into account the feasibility of the gating scheme, a *t_p_* = 20 nm thick lossless polysilicon layer with a permittivity of *ε_g_* = 3 [32,38] is presented between the two Topas layers, acting as a gating layer to control the conductivity of the patterned graphene sheet via electrostatic doping by tuning bias voltage *V_g_*. The bottom layer is a silver plate, which is regarded as a dispersive medium according to the Drude model [39]. The relative permittivity is given by *ε*(*ω*) = *ε_∞_* − *ω_p_*^2^/(*ω*^2^ + *i·ω_γ_*), where the high-frequency dielectric constant *ε_∞_*, the plasma frequency *ω_p_*, and the collision frequency *γ* are 1, 7.25 × 10^4^ cm^−1^ and 1.45 × 10^2^ cm^−1^, respectively. The top view of the absorber unit cell is shown in Figure 1b, where *p* is the period of the unit cell, *a* is the side length of the central square, *r* is the radius of the disk, and *b* is the width of the outer square loop of the patterned graphene sheet. The thickness of the graphene sheet, upper Topas dielectric spacer 1, polysilicon gating layer, lower Topas dielectric spacer 2, and silver plate are *t_g_*, *t_t_*_1_, *t_p_*, *t_t_*_2_, and *t_s_*, respectively. In this design, we set the dimension parameters as: *p* = 40 μm, *a* = 20$\sqrt{2}$ μm, *r* = 11 μm, *b* = 250 nm, *t_g_* = 0 nm, *t_t_*_1_ = 20 nm, *t_p_* = 20 nm, *t_t_*_2_ = 20.96 μm, and *t_s_* = 500 nm. In the finite element method based numerical simulations, the graphene sheet is modeled as an infinite-thin layer with 2D surface impedance *Z_g_* = 1/*σ_g_* [38]. Periodic boundaries in both *x*- and *y*-directions, and Floquet ports in the *z*-direction are assigned to the unit cell. The absorbance of this variety of absorbers can be calculated by the formula of *A* (*ω*) = 1 − *T*(*ω*) − *R*(*ω*), where transmission *T*(ω) = |*S*_21_|^2^ and reflectance *R*(ω) = |*S*_11_|^2^ [6,20]. In addition, it is worth mentioning that the curved edge and nanoscale effect of the graphene can be neglected because the narrowest width of the graphene is much larger than 50 nm [38,40], and the hybrid square/disk/loop patterned graphene sheet can be produced through state-of-the-art graphene nanofabrication techniques [41].

## 3. Results and Discussion

**Figure 2:**
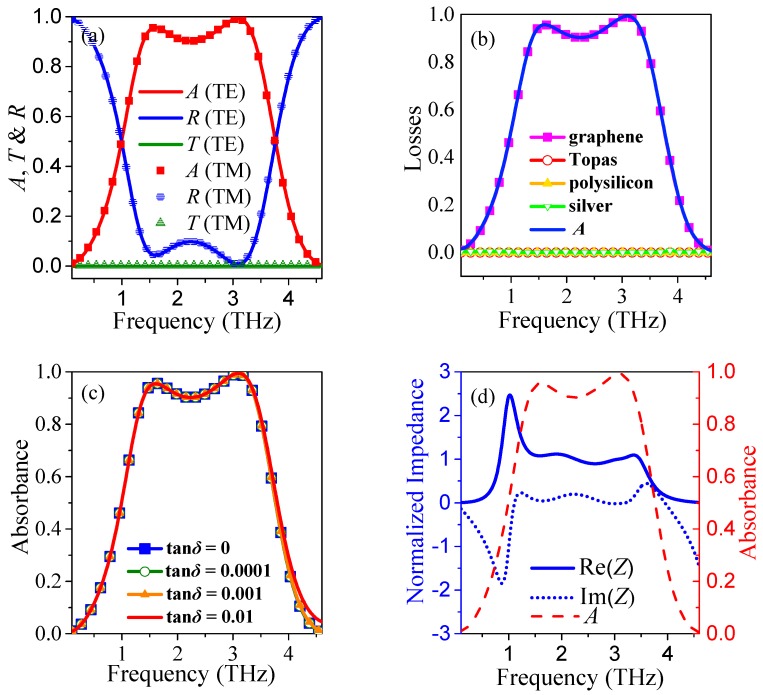
(**a**) Simulated reflection (*R*), transmission (*T*), and absorbance (*A*) of the proposed absorbers for both TE and TM polarizations at *µ_c_* = 0.7 eV under normal terahertz incidence; (**b**) Simulated surface and volume losses of the different materials of the proposed absorber; (**c**) Absorption spectra of the proposed absorber with different loss tangents tan *δ* = 0, 0.0001, 0.001, and 0.01 for both Topas and polysilicon; (**d**) Retrieved effective impedance (*Z*) of the proposed absorbers, where the blue solid curve and the blue dash-dot curve represent the Re(*Z*) and Im(*Z*), respectively, and the red dash curve is the absorption spectrum that is presented for contrast.

First, we study the absorption properties of the proposed polarization insensitive broadband terahertz absorber with a hybrid square/disk/loop patterned graphene under normal incidence. The Fermi level and relaxation time of the graphene are initially assumed to be *μ_c_* = 0.7 eV and *τ* = 0.1 ps. In this structure, the broadband terahertz surface plasmon resonance absorption will be enhanced by this patterned graphene sheet. Because the transmission is completely suppressed by the bottom silver layer, the maximum absorption of the absorber can be achieved when the broadband impedance matching condition of the terahertz incidence is satisfied. Figure 2a shows the transmission *T*, reflectance *R,* and absorbance *A* of the proposed absorber for both normal incident TE and TM polarized THz waves. As is clearly shown in this figure, the *T*, *R*, and *A* spectra for both of the polarizations of the absorber are perfectly matched, indicating good polarization-insensitive property, which is expected from the designed axisymmetric structure. The absorber has broadband absorption with a 90% absorbance bandwidth of 2.02 THz, from 1.38 THz to 3.4 THz, for both polarizations. The fractional bandwidth, the absolute bandwidth with respect to the central frequency, is about 84.5%. Figure 2b shows the surface and volume losses of the different material layers of the proposed absorber. Owing to the volume losses of both Topas and polysilicon are zero, as well as the silver layer acts as a nearly perfect reflector, the absorbance *A* is almost equal to the surface loss of the hybrid-patterned graphene. To further investigate the influence of the losses from dielectric spacers on the terahertz absorption, different loss tangents tan *δ* = 0, 0.0001, 0.001, and 0.01 are simultaneously assigned to the Topas and polysilicon in simulations, as shown in Figure 2c. It is observed that lossy dielectric spacers with tan *δ* ≤ 0.01 only result in a negligible absorbance enhancement. This is because the EM fields are extremely confined around the graphene layer, and almost all terahertz absorption results from the graphene layer. The absorption phenomenon can be interpreted through the effective medium theory [42]. The effective impedance *Z* (normalized to the impedance of free space) of the absorber can be retrieved from the complex frequency dependent reflection and transmission coefficients, as shown in Figure 2d. The blue solid curve and blue dash-dot curve represent the real part Re(*Z*) and the imaginary part Im (*Z*) of the effective impedance *Z*, respectively. The results reveal that Re(*Z*) is close to 1 and Im (*Z*) is close to 0 between 1.38 THz and 3.4 THz, implying that *Z* nearly matches the impedance of free space. Therefore, a broadband absorption with high absorbance is achieved owing to the small reflectance and complete lack of transmission of the absorber in that frequency range.

**Figure 3:**
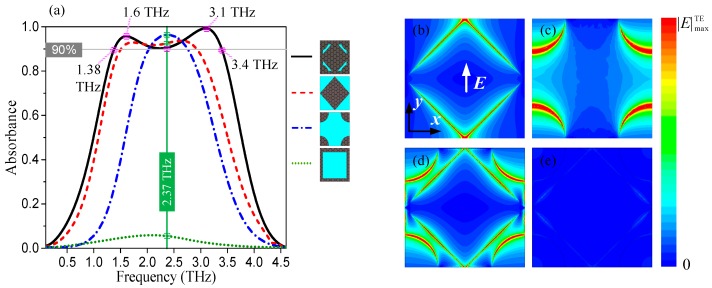
(**a**) The absorption spectra for the absorbers based on the patterned graphene unit cell with only square (red dash), four quarter-disk (blue dash-dot), square-loop (green dot), and the proposed hybrid square/disk/loop (black solid) resonators, under the same dimension parameters shown in Figure 1; (**b**), (**c**), and (**d**) are the normalized |*E*| distributions for TE polarization of the absorbers with only square, disk, and hybrid patterned graphene, respectively, on the graphene plane with high absorption at 2.37 THz; (**e**) is the normalized |*E*| distributions for TE polarization of the proposed absorber on the graphene plane with low absorption at 4.5 THz.

To investigate the broadband terahertz absorption property, we simulate and compare the absorption spectra and electric field distributions of the absorbers with different graphene patterns. Here, we consider the absorbers based on a patterned graphene unit cell with only one square resonator in the center, four quarter-disk resonators in the corners, a square-loop resonator, and the proposed hybrid square/disk/loop resonator (regarded as a combination of the aforementioned three structures) with the same dimension parameters as those shown in Figure 1. The absorption spectra of these four structures are illustrated in Figure 3a. The simulated results reveal that the 90% terahertz absorbance absolute (fractional) bandwidths of the square resonator (red dash) and the four square-disk resonators (blue dash dot) are 1.41 THz (64%), from 1.51 THz to 2.92 THz, and 0.72 THz (30%), from 2.03 THz to 2.75 THz, respectively, for the TE polarizations under normal incidence. However, owing to the very small strip width of *b*, the surface plasmon resonance of the square-loop-resonator occurs at a frequency much higher than the concerned absorption frequency range, and consequently, the absorption of the absorber based on square-loop resonators below 5 THz (green dot) is very small. By introducing a hybrid square/disk/loop patterned graphene resonator, the 90% absorbance bandwidth (fractional bandwidth) of the proposed absorbers can be significantly increased to 2.02 THz (84.5%), ranging from 1.38 THz to 3.4 THz; see the black solid curve as shown in Figure 3a. This electrically connected hybrid-patterned graphene structure also ensures the convenience of tuning via electrostatic doping of graphene. Furthermore, the electric distributions of the surface plasmon resonance for TE polarization on the graphene sheet in these structures under normal incidence are displayed in Figure 3b–e. Figure 3b–d illustrate the normalized |*E*| distributions for TE polarization of the absorbers that are based on the only square, disk and hybrid patterned graphene, respectively, on the graphene plane at a center frequency of 2.37 THz. It is clearly observed that the terahertz surface plasmon resonances are strongly bounded to the graphene edges at the frequency of 2.37 THz for different resonators, resulting in high terahertz absorbance over 90%. In particular, the surface plasmon resonance appears in more areas, including the edges of the square, disks, and loop of the proposed absorber. Figure 3e shows the |*E*| distributions of the proposed absorber on the graphene plane at 4.5 THz. The normalized |*E*| distributions that are shown in Figure 3b–e match the absorption characteristics shown in Figure 3a well. Notably, because of the designed axisymmetric structure, the electric field distributions for TM polarization of these structures are the same as that of TE polarization but with 90° azimuth rotation.

**Figure 4:**
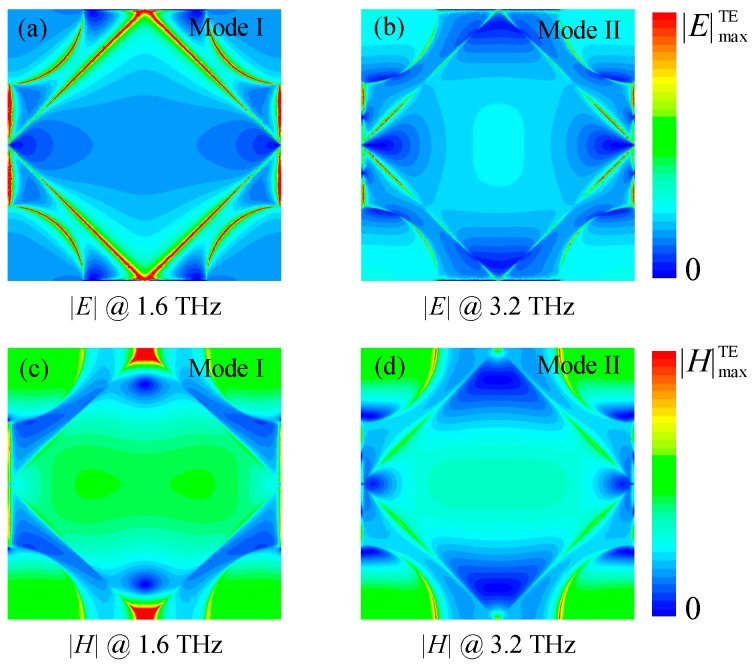
(**a**–**d**) The electric and magnetic field distributions at the resonance frequencies of 1.6 THz and 3.2 THz on the hybrid-patterned graphene layer of the proposed absorber, respectively.

**Figure 5:**
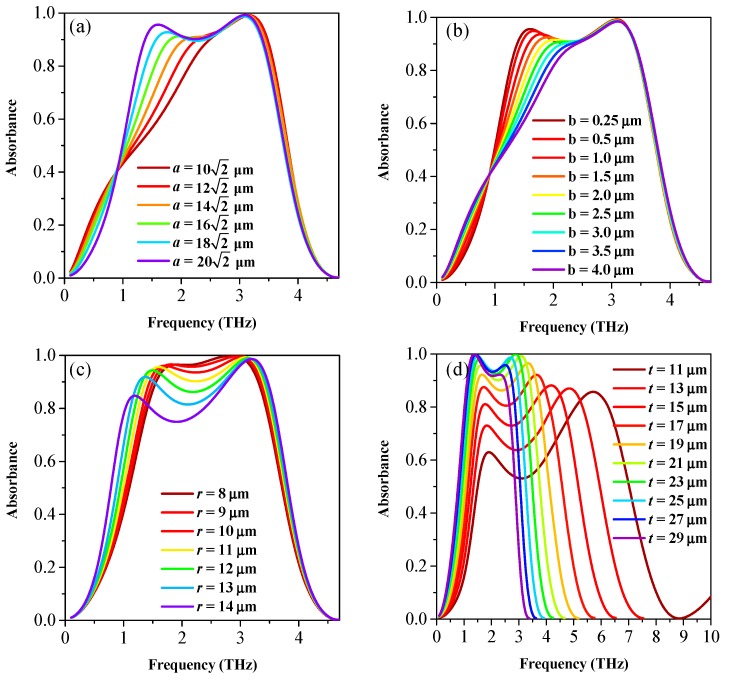
The absorption spectra as a function of the geometric parameters *a*, *b*, *r*, and *t* are displayed in (**a**), (**b**), (**c**), and (**d**), respectively. Particularly, in (**a**), we reduce the *a* while increasing *b* to maintain the $\sqrt{2}$*a* + 2*b* = 40 μm and to keep the electric continuity of the hybrid-patterned graphene; in (**b**–**d**), only one single parameter *b*, *r*, or *t* is changing while keeping the other parameters fixed.

To get more insight into the physical origin of broadband absorption, we plot the normalized |*E*| and |*H*| field distributions of graphene in-plane resonant mode peaks at 1.6 THz and 3.2 THz, as shown in Figure 4. Here, according to Ref. [43], we assign these two modes around 1.6 THz and 3.2 THz to be the laterally-propagating surface mode I and mode II, respectively. It is clear that the |*E*| and |*H*| distributions of these modes are distributed at a different part of the hybrid patterned graphene area. The absorbance can be enhanced when the impedance is matched at the resonance frequency of these laterally-propagating modes, and its spectral bandwidth can be expanded using the two optimized modes. To demonstrate these characteristics, we simulate the absorption spectra as a function of the geometric parameters *a*, *b*, *r*, and *t* (the total dielectric spacer thickness). In the calculations, the dimensions are fixed as *p* = 40 μm, *a* = 20$\sqrt{2}$ μm, *r* = 11 μm, *b* = 250 nm, *t_g_* = 0 nm, *t_t_*_1_ = 20 nm, *t_p_* = 20 nm, *t_t_*_2_ = 20.96 μm, and *t_s_* = 500 nm, if not specifically indicated. Figure 5a–c generally show that the parameters of the patterned graphene have a significant influence on the absorbance around the resonant frequency *f*_I_ of mode I while having little influence around the resonant frequency *f*_II_ of mode II. Particularly, the higher *f*_I_ and the lower absorbance *A* of mode I can be observed with *a* and *b* increasing, while, both *f*_II_ and *A* of mode II decrease with *r* increasing. Furthermore, Figure 5d clearly shows that, as the *t* decreases, the *f*_I_ slightly increases and the *f*_II_ greatly increases with the lowering Q factors. In this way, the bandwidth of the absorber can be significantly expanded by overlapping these two modes with relatively farther-apart resonant frequencies. In this study, we choose the geometric parameters with *a* = 20$\sqrt{2}$ μm, *b* = 250 nm, *r* = 11 μm, and *t* = 21 μm to obtain the optimum 90% absorbance bandwidth property of the absorber.

**Figure 6:**
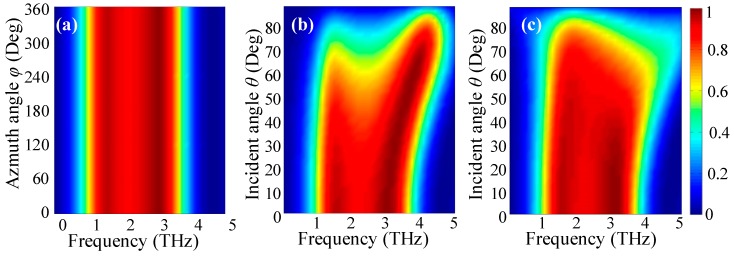
The simulated absorption spectra of the proposed absorber as functions of frequency and the terahertz wave polarization angle *φ* (**a**), as well as the frequency and the oblique incident angle *θ* for both TE polarization (**b**) and TM polarization (**c**).

Then, we further investigate the terahertz absorption behavior dependence on the angle of the polarizations and the angle of incidence. To eliminate polarization sensitivity, the absorber is carefully designed by arranging the individual disk and square and loop resonators axisymmetrically into the hybrid patterned graphene. Figure 6a shows the dependence of absorption spectra on the azimuthal angle (polarization angle) *φ* at normal incidence (*θ* = 0°). It is clear that the proposed broadband absorber has absolute polarization insensitivity for all polarizations with *φ* ranging from 0° to 360° for the terahertz waves at normal incidence. As we know, the omnidirectionality is another important property for an absorber. To examine this feature, the simulated absorbance of the absorber as functions of frequency and the oblique angle of incidence *θ* for both TE and TM polarized terahertz waves are depicted in Figure 6b,c, respectively. It is found that the proposed absorber has stable absorption bandwidth with the incident angle *θ* ranging from 0° to 60° for both polarizations. This is because the absorption properties of the absorber are mainly related to the tightly confined surface plasmon resonances, and the bandwidth is mainly decided by the hybrid disk, square, and loop patterned graphene structure, while being less dependent on the angle of incidence. It is also observed that the peak absorbance decreases gradually as the incident angle increases for both polarizations, and the peak absorbance of TM polarization decreases even faster than that of the TE polarization. That is owing to fact that the orientation of the electric field for the electric dipole resonance in the patterned graphene changes for TM polarization, while that remains for TE polarization when the incident angle is varied. Moreover, owing to the parasitic resonances that occur at a large angle of the incidence, slightly blue-shifts of the absorption spectra can also be observed. Nevertheless, the broadband absorption effect is still very strong, even at a large oblique incident angle; when *θ* = 60°, the absorbance remains more than 60% with bandwidth 2.7 THz from 1.3 THz to 4 THz for both TE and TM polarizations.

**Figure 7:**
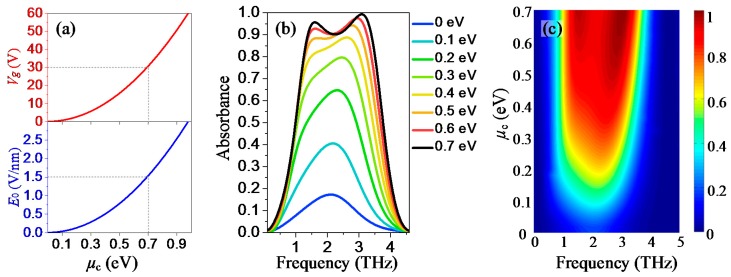
(**a**) displays the relationship between *µ_c_* and *E*_0_, *µ_c_* and *V*_g_; (**b**) and (**c**) show the simulated absorption spectra curves and colormap with different Fermi levels from 0 eV to 0.7 eV for both TE and TM polarizations, respectively.

Finally, the absorption tunability of the proposed absorber is also studied. As illustrated in Figure 1a, gate voltage *V_g_* is applied to the graphene-Topas spacer-polysilicon parallel plate capacitor to control the conductivity of graphene via the electrostatic doping effect. The relationship between *V_g_* and Fermi level *μ_c_*, as well as *E*_0_ and *μ_c_* are given by the formula [35]: $V_{g}=E_{0}\cdot t_{t1}=\frac{e\cdot n_{s}}{C}=\frac{e\cdot n_{s}}{\varepsilon_{r}\varepsilon_{0}/t_{t1}}$, where $n_{s}=\frac{2}{\pi \hbar^{2}v_{F}^{2}}\int_{0}^{\infty }\xi [f_{d}(\xi ,\mu_{c},T)-f_{d}(\xi +2\mu_{c},\mu_{c},T)]d\xi$. From these equations, the *µ_c_*, *E*_0_ Curve and the *µ_c_*, *V*_g_ curve are plotted in Figure 7a. It is found that the required values of *E_0_* and *V_g_* for achieving the *µ_c_* of 0.7 eV are around 1.5 V/nm and 30 V, respectively, which are achievable in the experimental work. By taking advantage of the graphene conductivity tunability, the absorption tuning of the proposed absorber can be achieved. The absorption tunability as functions of frequency and the Fermi level ranging from 0 to 0.7 eV are depicted in Figure 7b,c. It is observed that the peak absorbance can be adjusted from lower than 20% to higher than 90% while maintaining the operating frequency by changing the Fermi level from 0 eV to 0.7 eV, corresponding to the gate voltage from 0 V to 30 V. Therefore, the proposed absorber is an excellent tunable broadband terahertz absorber with absolute polarization insensitivity and almost omnidirectionality, which can be engineered for various applications in optoelectronic devices, including broadband spatial amplitude modulators, sensors, and detectors in the terahertz region.

## 4. Conclusions

In this study, we have demonstrated an actively tunable polarization-insensitive broadband terahertz absorber based on a hybrid-patterned graphene metasurface. The single-layered graphene with periodically electric-connected hybrid-patterned square/disk/loop structures can effectively enhance the terahertz absorption bandwidth. The results show that the absorber has over 2 THz (with a fractional bandwidth of 84.5%) for 90% absorbance and absolute polarization insensitivity under normal incidence. The absorber also demonstrates near omnidirectionality with 2.7 THz bandwidth for 60% terahertz absorbance for the wide incident angle, ranging from 0° to 60° for both TE and TM polarizations. By introducing a simple polysilicon gating layer beneath the graphene layer, the absorber absorbance can be flexibly tuned from more than 90% to lower than 20% by changing the Fermi level from 0.7 eV to 0 eV. Benefitting from these promising properties, the proposed absorber may have great potential applications in terahertz sensing, detecting, imaging, cloaking, and optoelectronic devices.
